# Neutrophil-to-Lymphocyte, Lymphocyte-to-Monocyte, and Platelet-to-Lymphocyte Ratios: Prognostic Significance in COVID-19

**DOI:** 10.7759/cureus.12622

**Published:** 2021-01-11

**Authors:** Shivakumar BG, Siddharth Gosavi, Amogh Ananda Rao, Shashank Shastry, Sharanya C Raj, Anirudha Sharma, Ashutosh Suresh, Rakesh Noubade

**Affiliations:** 1 Internal Medicine, Jagadguru Jayadeva Murugarajendra Medical College, Davangere, IND

**Keywords:** covid-19, prognostic markers, complete blood count

## Abstract

Background and objective

Neutrophils are primarily responsible for activating the immune system, and systemic inflammation destroys CD4+ T lymphocytes and increases suppressor CD8+ T lymphocytes, thereby leading to an increased neutrophil-to-lymphocyte ratio (NLR). An increase in the apoptosis of lymphocytes leads to lymphopenia and elevated thrombopoietin (THPO) promotes megakaryocyte production. The reflections of these inflammatory changes can be vital in gauging the progression of the disease.

This study aimed at examining the prognostic value of normal and derived neutrophil-to-lymphocyte, lymphocyte-to-monocyte, platelet-to-lymphocyte, and mean platelet volume (MPV)-to-platelet count ratios in patients with coronavirus disease 2019 (COVID-19).

Methodology

This was a retrospective cross-sectional study conducted in the wards of Chigateri General Hospital, Davangere for a period of two months. Complete blood count was ordered for all patients at the time of admission along with confirmation of the disease by reverse transcription-polymerase chain reaction (RT-PCR).

Results

The final study population consisted of 100 patients. The mean age of patients who survived (43 years) was significantly lower than the mean age of non-survivors (59.1 years), with a p-value of <0.001. NLR was raised in 60% of the population and was significantly higher in patients who survived the disease, with a p-value of 0.004. The platelet-to-lymphocyte ratio (PLR) also followed a similar trend with a p-value of 0.017. Even though the lymphocyte-to-monocyte ratio (LMR) also mimicked the trend, the statistical association was not significant (p-value: 0.09). The derived NLR and MPV-to-platelets ratios were not found to be significantly associated with mortality in this study.

Discussion

Younger patients had better clinical outcomes in our study population compared to the geriatric age group. A significant correlation between LMR and mortality was observed when a cut-off of 2.5 was considered as a differentiating benchmark.

Conflicting trends were observed in NLR and PLR in our study; however, LMR was in accordance with most other studies. The phase that a patient is in with regard to the natural history of the disease also influences the blood cell ratios. Nonetheless, all three ratios can be used as crucial screening and prognostic tools as they are readily available with the help of a complete hemogram. This is an investigation modality that is widely accessible even in remote areas and resource-limited settings.

Conclusion

These hematological ratios can facilitate in categorizing the disease severity and progression in patients, thereby enabling us to make appropriate and informed clinical decisions. Since the second wave of the novel coronavirus is on the verge of arrival, it is imperative to channel resources for the patients early in their disease course to ultimately prevent complications and reduce mortality.

## Introduction

Neutrophils constitute the majority of the leukocytes and are primarily responsible for activating the immune system by migrating from the venous system. Free oxygen radicals that can damage the nuclear material of the cell are thereby released. Viral antigens are exposed and cell-specific and humoral immunities are stimulated by an antibody-dependent cell-mediated cell. There is a growing interaction with molecules like vascular endothelial growth factor (VEGF), interleukin-6, interleukin-8, tumor necrosis factor-alpha (TNF-α), interferon-gamma, and granulocyte colony-stimulating factor. VEGF-A and VEGF-C are particularly notorious in the novel coronavirus, severe acute respiratory syndrome coronavirus-2 (SARS-CoV-2) [1]. The immune response is massively dependent on lymphocytes. On the other hand, systemic inflammation destroys CD4+ T lymphocytes and increases suppressor CD8+ T lymphocytes, thereby leading to an increased neutrophil-to-lymphocyte ratio (NLR). Formerly, NLR was mainly used in oncological conditions, autoimmune diseases, and bacteriological infections [2,3]. However, in a study conducted by Yang et al., it was found to be an independent prognostic factor in patients with coronavirus disease 2019 (COVID-19). This reinforces the belief in the relationship between hyper-inflammation and SARS-CoV-2 [4]. In another study conducted by Ciccullo et al, an NLR of greater than four was seen as a predictor of the admission of COVID-19 patients to the intensive care unit. The duration of hospital stay was prolonged, and the time lag for nucleic acid results to become negative was increased. It is a valuable tool for screening critically ill patients with confirmed SARS-CoV-2 infection, as it can contribute to the evaluative acumen of the physician [5].

T helper cells induce the production of cytokines such as interleukin-17 through the nuclear factor kappa-light-chain-enhancer of activated B cells (NF-kB) signaling pathway, leading to increased aggregation of monocytes. SARS-CoV-2 infects circulating immune cells and increases apoptosis of lymphocytes, leading to lymphopenia. A lower ratio of circulating lymphocytes to monocytes (LMR) predicts severe and extremely severe COVID-19 as the clearance of the virus is delayed due to lymphopenia and also a decrease in CD4+ T cells. Both a rise or fall in lymphocyte levels is an extremely crucial prognostic indicator of mortality in COVID-19 [6].

Platelets play a crucial role in homeostasis, coagulation, vascular integrity maintenance, angiogenesis, innate immunity, and inflammatory response. In a state of inflammation, interleukin-6 promotes megakaryocyte production by stimulating an increase of thrombopoietin (THPO) level. Platelet count is a sensitive reflection of the body’s infection and inflammatory state. In a study conducted by Qu et al., it was found that platelets increased first and then decreased in several of their patients during treatment. Coronavirus invades bone stromal cells, leading to hematopoietic inhibition [7]. Also, mature megakaryocytes release platelets in the lung. Therefore, thrombocytopenia and lung damage go hand in hand. Extensive alveolar damage is also observed. Lung damage results in pulmonary endothelial injury, which in turn leads to activation, aggregation, and retention of platelets in the lung. It is followed by the formation of a thrombus, which leads to the depletion of platelets. The cytokine storm is responsible for worsening the inflammation in the patient as it causes increased secretion of Th2 cytokines that inhibit Th1 cytokines, such as interleukin-4 and interleukin-10. Tracheal intubation and deep vein catheterization are potential factors that affect platelet changes. Platelet-released platelet factor-4 can prevent agglutinin-A from inhibiting lymphocyte generation; activated platelets enhance lymphocyte adhesion to the endothelium, thereby promoting lymphocyte homing in endothelial veins and migration to inflammatory sites. It indicates both aggregation and inflammatory pathways, thereby showing the level of inflammation even during the course of treatment. This also correlated with an increase in the stay at the hospital. Therefore, the platelet-to-lymphocyte ratio (PLR) provides a reliable reflection of the extent of cytokine inflammation and can be employed for monitoring purposes in patients with COVID-19 [8].

The mean platelet volume (MPV)-to-platelet count ratio has been recently proposed as a prognostic marker in SARS-CoV-2. In inflammation, platelet production increases due to the increased synthesis of THPO, which is mediated through cytokines. MPV reflects the proliferation of megakaryocytes and platelet production in the bone marrow. There is an increased expression of young platelets in the bloodstream, leading to increased MPV. Decreasing platelet count forces the body’s immune system to stimulate megakaryocytes to produce a large number of platelets, thereby increasing MPV. Adverse prognosis is due to the increased oxidative stress, thrombosis, and apoptosis in activated platelets [9]. It symbolizes a higher risk of poor outcomes in COVID-19 patients.

These four ratios have been scrutinized in great detail in SARS-CoV-2 patients in other countries that have a higher financial and resource allocation for healthcare. However, in resource-limited settings or where a health facility is functioning beyond its capacity, investigations like interleukin-6, interleukin-10, and other special platelet tests cannot be routinely performed. Therefore, by employing a simple complete blood count, we aimed to study the importance of derived NLR along with the other four ratios in patients with SARS-CoV-2. We also aimed to investigate their relationship with mortality in patients with COVID-19.

## Materials and methods

This was a retrospective cross-sectional study conducted at the Chigateri General Hospital, Davangere, for a period of two months: July and August of 2020. We commenced the study after obtaining approval from the Institutional Ethics Committee.

**Figure 1:**
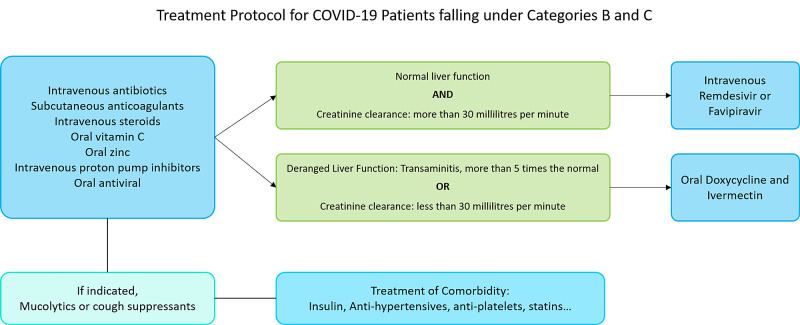
Treatment protocol for COVID-19 patients falling under Categories B and C Categories B and C were defined by the interim guidelines issued by the Ministry of Health and Family Welfare, Government of India COVID-19: coronavirus disease 2019

Cases were defined based on the interim guidelines issued by the Ministry of Health and Family Welfare, Government of India. Admitted patients with the respiratory rate exceeding 24 cycles per minute and oxygen saturation below 94% without oxygen supplementation (falling under Category B and Category C) were included in the study. A complete hemogram was ordered within one hour of admission, before the initiation of treatment. The diagnosis of COVID-19 was confirmed with reverse transcription-polymerase chain reaction (RT-PCR) for viral RNA. Patients receiving mechanical ventilation, patients on tocilizumab, pirfenidone, azathioprine, and cyclophosphamide, and those with a previous diagnosis of malignancy were excluded from the study. Patients with other febrile illnesses like dengue, malaria, leptospirosis, and rickettsial fevers were also excluded from the study. Figure 1 presents the treatment protocol followed in the care of all patients in the study population.

Various ratios that are known to have prognostic value and examined in this study are presented in Table 1. The laboratory records were collected and screened to match the criteria of our study. Data analysis was performed using IBM SPSS Statistics version 27 (IBM, Armonk, NY).

**Table 1:** Definition of prognostic markers

Parameter	Acronym	Formula
Neutrophil-to-lymphocyte ratio	N/L	(Absolute neutrophil count) / (absolute lymphocyte count)
Derived neutrophil-to-lymphocyte ratio	Derived N/L	(Absolute neutrophil count) / (total leukocyte count – absolute neutrophil count)
Lymphocyte-to-monocyte ratio	L/M	(Absolute lymphocyte count) / (absolute monocyte count)
Platelet-to-lymphocyte ratio	Plt/L	(Platelet count) / (absolute lymphocyte count)
Mean platelet volume-to-platelet count ratio	MPV/Plt	(Mean platelet volume) / (platelet count)

Results were presented as mean, standard deviation, and range values for continuous measurements and frequencies as numbers and percentages. The unpaired t-test was used to compare the means between any two groups. Whenever measurements displayed non-Gaussian distribution, the Mann-Whitney U test was used as an alternative to the t-test. Categorical data were analyzed by the chi-squared test. The odds ratio was used to compute the odds of occurrence of an event (mortality) using bio-marker. Diagnostic validity tests were also performed to assess the utility of each parameter. Receiver operator characteristic (ROC) curves were used to compare the different parameters. A p-value of 0.05 or less was considered statistically significant.

## Results

The final study population consisted of 100 patients. Table 2 presents the demographic description of the sample. The mean age was 47.1 ±14.8 years (range: 20-78 years). There were 57 males and 43 females were; 75 patients survived the viral disease, and 25 patients succumbed to the virus.

The mean age of patients who survived (43 years) was significantly lower than that of non-survivors (59.1 years), with a p-value of <0.001.

**Table 2:** Descriptive information of study subjects The mean age of patients who did not survive the disease was significantly higher (p<0.001) SD: standard deviation; HS: highly significant; NS: not significant

Demographic characteristics					
Number of cases		All cases	Non-survivors	Survivors	Non-survivors vs. survivors
		100	25	75	
Age (years)	Mean ±SD	47.1 ±14.8	59.1 ±11.5	43.0 ±13.6	t = 5.31; p<0.001 (HS)
	Range	20–78	40–78	20–71	
Sex	Male	57	13	44	X^2^ = 0.34; p = 0.56 (NS)
	Female	43	12	31	

**Figure 2:**
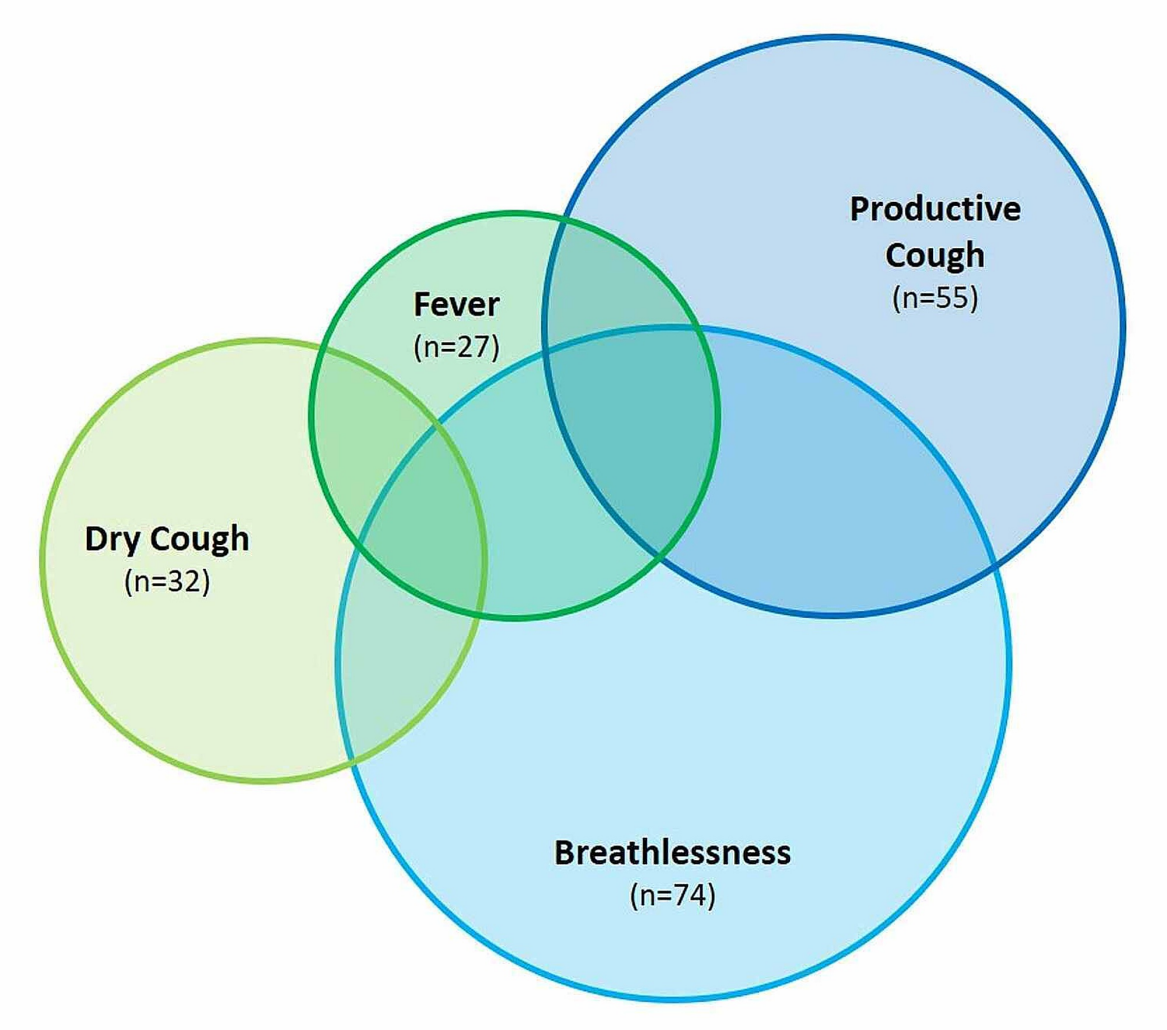
Representation of symptomatology

Figure 2 depicts the presenting complaints of all the patients. Breathlessness was the most frequent complaint, followed by productive and dry cough, and fever. The duration of symptoms ranged from one to five days. Table 3 presents a comparison of the symptoms between survivors and non-survivors.

**Table 3:** Comparison of symptoms between survivors and non-survivors

Symptom	Non-survivors (n=25)	Survivors (n=75)
Fever	10	15
Dry cough	9	23
Productive cough	12	43
Breathlessness	17	57

**Figure 3:**
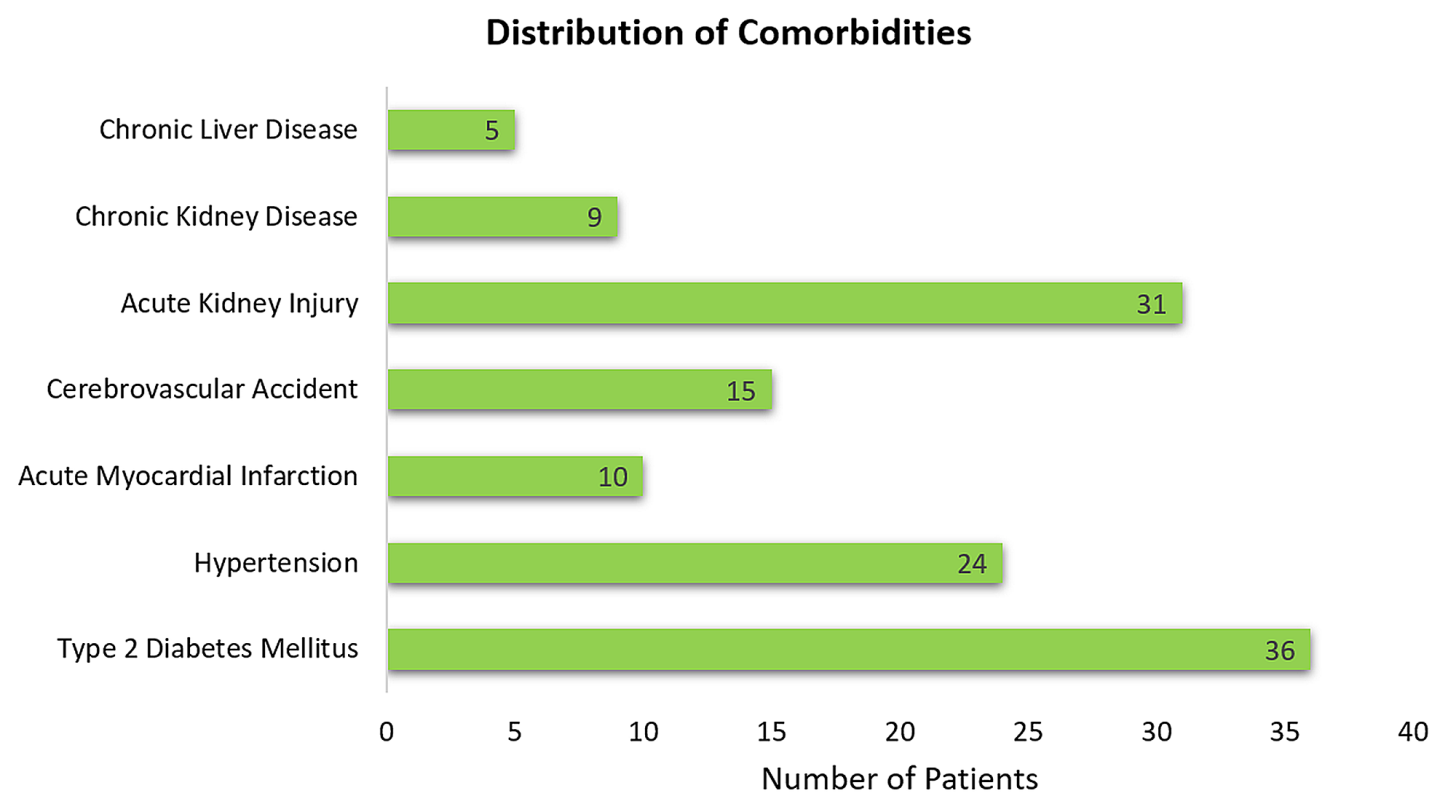
Distribution of comorbidities in the study population

Figure 3 documents the presence of comorbidities in the study population. Diabetes mellitus was the most common accompanying morbidity, followed by hypertension and other conditions. Table 4 provides a comparison with respect to comorbidities between patients who survived the disease and those who succumbed to the disease.

**Table 4:** Comparison of comorbid conditions between survivors and non-survivors

Comorbidity	Non-survivors	Survivors
Type 2 diabetes mellitus	19	17
Hypertension	5	19
Acute myocardial infarction	5	5
Cerebrovascular accident	3	12
Acute kidney injury	4	27
Chronic kidney disease	4	5
Chronic liver disease	1	4

Table 5 summarizes the distribution of various cell lines included in the calculation of prognostic parameters investigated in this study.

**Table 5:** Descriptive statistics of cell lines used to compute prognostic parameters

Cell line	Mean	Standard deviation	Median	Minimum	Maximum
Total leukocyte count (cells/cumm)	9,775	5,698.1	8,300	800	31,400
Platelet count (cells/cumm)	22,849	9,646.2	22,200	2,300	46,700
Absolute neutrophil count (cells/cumm)	7,063.1	5,089.8	5,457.6	247.5	29,257
Absolute lymphocyte count (cells/cumm)	1,471.3	905.7	1,438.8	102.4	4,647.2
Absolute monocyte count (Cells/cumm)	884.5	745.1	671.5	22.5	3,799

Table 6 presents the descriptive statistics of the prognostic parameters for correlation with disease outcomes. The NLR was raised in 60% of the population.

**Table 6:** Descriptive statistics on test measurements SD: standard deviation; N/L: neutrophil-to-lymphocyte ratio; L/M: lymphocyte-to-monocyte ratio; Plt/L: platelet-to-lymphocyte ratio; MPV/Plt: mean platelet volume-to-platelet count ratio

Parameter	Mean ±SD	Median	Minimum	Maximum
Derived N/L	3.49 ±3.02	2.69	0.45	20.28
N/L	7.9 ±8.9	4.1	0.99	39.71
L/M	3.06 ±4.15	2.07	0.16	27.71
Plt/L	27.0 ±42.6	18.5	1.4	300.8
MPV/Plt	0.00052 ±0.0005	0.0004	0.0002	0.0037

Table 7 compares the prognostic markers between survivors and non-survivors. The NLR was significantly higher in patients who survived the disease, with a p-value of 0.004. PLR also followed a similar trend with a p-value of 0.017. Even though LMR also mimicked the trend, the statistical association was not significant (p-value: 0.09).

**Table 7:** Comparison of test parameters between non-survivors and survivors N/L and Plt/L differ significantly between survivors and non-survivors, with p-values of 0.004 and 0.017 respectively SD: standard deviation; N/L: neutrophil-to-lymphocyte ratio; L/M: lymphocyte-to-monocyte ratio; Plt/L: platelet-to-lymphocyte ratio; MPV/Plt: mean platelet volume-to-platelet count ratio; S: significant; NS: not significant

Parameter	Non-survivors (n=25)		Survivors (n=75)		Non-survivors vs. survivors	
	Mean	SD	Mean	SD	t	P-value
Derived N/L	2.65	1.93	3.77	3.27	-1.61	0.11 (NS)
N/L	4.87	3.7	8.88	3.84	2.96	0.004 (S)
L/M	2.17	2.13	3.35	4.61	-	0.09 (NS)
Plt/L	15.43	9.44	30.52	48.21	-	0.017 (S)
MPV/Plt	0.00028	0.00046	0.00047	0.00072	-	0.14 (NS)

The correlation of the prognostic parameters in relation to the clinical outcome is presented in Table 8. The cut-off value for each parameter was considered as a suitable value around the median, which showed high sensitivity and accuracy in the prediction of mortality. The statistical correlation between the clinical outcomes and the cut-off values has been shown.

**Table 8:** Ratio-wise distribution of cases and their significance in differentiating the final outcome N/L: neutrophil-to-lymphocyte ratio; L/M: lymphocyte-to-monocyte ratio; Plt/L: platelet-to-lymphocyte ratio; S: significant

Ratio-wise distribution of cases and their significance								
Test parameter	Cut-off value	Non-survivors (n=25)		Survivors (n=75)		Non-survivors vs. survivors		Odds ratio (95% CI)
		Number	%	Number	%	X²	P-value	
N/L	≤4.7	18	72.0	37	49.3	3.89	0.049 (S)	2.6 (1.0–7.1)
	>4.7	7	28.0	38	50.7			
L/M	≤2.5	22	88.0	41	54.7	8.94	0.003 (S)	6.1 (1.7–22.1)
	>2.5	3	12.0	34	45.3			
Plt/L	≤20.0	20	80.0	43	57.3	4.13	0.04 (S)	3.0 (1.0–8.8)
	>20.0	5	20.0	32	42.7			

**Figure 4:**
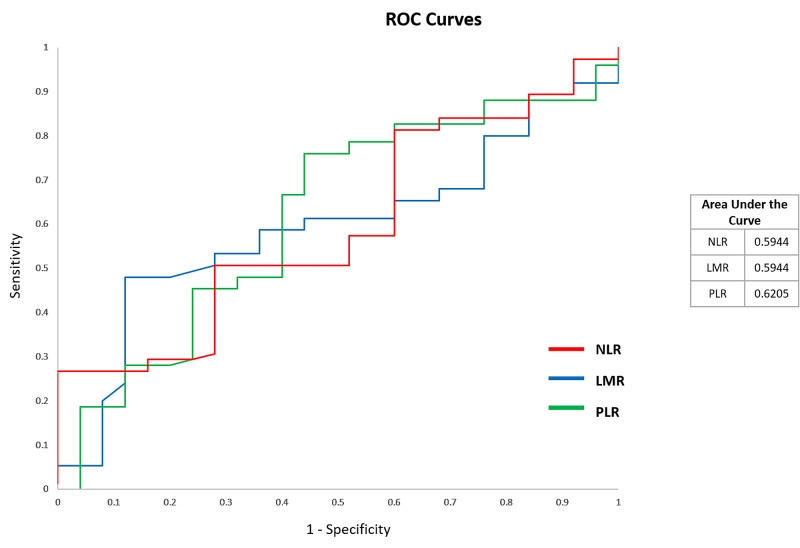
ROC curve and area under the curve ROC: receiver operator characteristic; NL: neutrophil-to-lymphocyte; LM: lymphocyte-to-monocyte; PltL: platelet-to-lymphocyte

Diagnostic test validity of NLR, LMR, and PLR was analyzed regarding the prediction of mortality of COVID-19 using a ROC curve (Table 9, Figure 4). These tests have a high sensitivity and high negative predictive value. These parameters can be used to rule out the possibility of death when these values are high.

**Table 9:** Diagnostic validity tests for predicting mortality using various significant parameters N/L: neutrophil-to-lymphocyte ratio; L/M: lymphocyte-to-monocyte ratio; Plt/L: platelet-to-lymphocyte ratio; PPV: positive predictive value; NPV: negative predictive value

Parameters	N/L	L/M	Plt/L
	≤4.7	≤2.5	≤20.0
Sensitivity	72%	88%	80%
Specificity	51%	45%	43%
PPV	33%	35%	32%
NPV	84%	92%	87%
Accuracy	56%	56%	52%

## Discussion

Younger patients had better clinical outcomes in our study population compared to the geriatric age group. The difference in mean age was 16 years between survivors and non-survivors. This observation is consistent with other studies that have implicated old age to be a factor contributing to worse clinical course and mortality [10,11].

NLR was higher than normal in the majority of the patients. However, it was 1.8 times higher among survivors in our study. This is in stark contrast with the trend observed in a study conducted by Usul et al., wherein NLR was was found to be significantly high in critically ill COVID-19 patients compared to the control population [12]. LMR was also higher in survivors but the association was not statistically significant. The LMR decreased in non-survivors in our study, which is in agreement with studies conducted by Lissoni et al. and Zhang et al. [13,14]. However, a significant correlation was observed with mortality when a cut-off of 2.5 was considered as a differentiating benchmark. PLR was twice as high among patients who survived compared to the non-survivors in our study. This observation also was in contrast with a study conducted by Zhao et al., wherein the PLR increased significantly in critically ill patients [15]. The derived NLR and MPV-to-platelets ratio were not found to be significantly associated with mortality in this study.

Conflicting trends were observed in NLR and PLR in our study; however, LMR was in accordance with most other published studies. The point of observation in our study was that at the time of admission and follow-up, investigations were not performed in all patients due to financial and logistic constraints. The phase that a patient is in with regard to the natural history of the disease also influences the blood cell ratios. Since the objective was to derive prognostic insights from the ratios, all the investigations were ordered at the time of admission to the hospital.

All three ratios can be used as crucial screening and prognostic tools as they are readily available with the help of a complete hemogram. This is an investigation that is widely accessible even in remote areas and resource-limited settings. This is a cost-effective tool in situations where logistics and finances are constrained. In our study, all three ratios were found to have high sensitivity and high negative predictive values, helping to gauge the progression of disease early in its course.

Limitations

This study has some limitations. Serial investigations could not be performed due to financial and logistical constraints related to the patients. The sample size was small and, hence, further, larger studies are warranted. Patients could not be followed up after discharge for persisting symptoms or post-COVID sequelae as the majority were anxious and apprehensive about returning to the hospital.

## Conclusions

These hematological ratios that we discussed in our study can facilitate in categorizing the disease severity and progression in patients, thereby enabling us to make appropriate and informed clinical decisions. Since the second wave of the novel coronavirus is on the verge of arrival, it is imperative to channel resources for the patients early in their disease course to ultimately prevent complications and reduce mortality.
